# Trends in hypertensive heart disease-related mortality among older adults in the USA: a retrospective analysis from CDC WONDER between 1999 and 2020

**DOI:** 10.1186/s43044-025-00622-6

**Published:** 2025-03-04

**Authors:** Muhammad Sameer Arshad, Zoaib Habib Tharwani, F. N. U. Deepak, Ali Abdullah, Rohet Kumar, Riteeka Kumari Bhimani, Raja Subhash Sagar, Parshant Dileep Bhimani, Adarsh Raja, Om Parkash, Muhammad Umer Sohail, Muhammad Mustafa Memon

**Affiliations:** 1 Dow Medical College, Dow University of Health Sciences, Karachi, Pakistan; 2 Shaheed Mohtarma Benazir Bhutto Medical College Lyari, Karachi, Pakistan; 3 Jinnah Sindh Medical University, Karachi, Pakistan; 4 Liaquat University of Medical & Health Science, Jamshoro, Pakistan; 5 Department of Medicine, Drexel University College of Medicine, Philadelphia, PA USA; 6 Department of Medicine, Rochester General Hospital, Rochester, NY USA

## Abstract

**Background:**

While hypertensive heart disease (HHD) has been widely studied, this study uniquely examines the impact of the COVID-19 pandemic on HHD mortality trends, which has not been thoroughly explored in the current literature. The pandemic’s effects on healthcare access, economic instability, and social isolation present new challenges and opportunities for understanding HHD mortality among the elderly.

**Results:**

Age-adjusted mortality rates (AAMRs) increased overall between 1999 and 2020, from 36.7 to 133.9 per 100,000 people, according to analysis. The data on AAMRs indicated a consistent rise from 1999 to 2017, with a notable uptick from 2017 to 2020. An investigation based on gender revealed that older men had a consistently higher AAMR than older women. The biggest AAMRs were found among the non-Hispanic (NH) Black or African-American population, according to variations in AAMR based on race and ethnicity. Geographic differences between states revealed that compared to Nebraska, Oregon, North Dakota, Maine, and Minnesota, the District of Columbia, Oklahoma, Nevada, Vermont, and Mississippi had substantially higher AAMRs. The West, Northeast, and Midwest were in second place with a continuously higher AAMR, followed by the South. Furthermore, compared to non-metropolitan areas, metropolitan areas had a higher AAMR.

**Conclusion:**

The importance of including demographic and geographic factors in public health planning and interventions is highlighted by these findings, which provide insightful information on mortality trends associated with HHD in the elderly.

**Supplementary Information:**

The online version contains supplementary material available at 10.1186/s43044-025-00622-6.

## Background

Hypertensive heart disease (HHD) is a major global health concern that impairs quality of life and causes a number of consequences. According to estimates, 10.8 million people worldwide suffer with HHD, which also causes 25% of all deaths in the USA. Heart disease continues to be the leading cause of death in the USA, contributing to 25% of all fatalities [1, 2].

A variety of anatomical and functional problems in the left ventricle (LV), such as LV hypertrophy brought on by chronic hypertension, are indicative of heart failure [3–5]. Compared to other cardiovascular disorders including ischemic heart disease (IHD), heart failure (HF) and cardiac arrhythmias usually develop later in life from HHD [6]. In the USA, 48.1% of adults (119.9 million) have hypertension, and just roughly one in four persons (22.5 percent, or 27.0 million) have their hypertension adequately treated [7]. The US spends, on average, $131 billion a year on this illness over a 12-year period, from 2003 to 2014 [8, 9].

Given that the risk of HHD rises with age, the effects of an aging global population are amplified. Although quantitative data are limited, studies have suggested that a variety of factors, including environmental impacts, healthcare accessibility, and demographic features, can alter death rates due to HHD [10]. Thus, from 1999 to 2020, we aimed to investigate the temporal patterns in HHD-related mortality among persons in the USA who were 55 years of age or older.

## Methods

### Population and study setting

For this study, we collected data from death certificates using the Wide-Ranging Online Data for Epidemiologic Research (CDC WONDER) database from the Centres for Disease Control and Prevention. The frequency of hypertensive heart disease (HHD)-related deaths in the senior population was examined in this study between 1999 and 2020. The 10th iteration of the International Classification of Diseases and Related Health Problems (ICD-10) code, or I11, was used to analyze and publish these death rates. The Multiple Cause-of-Death Public Use registration provided the death certificates from which the causes of death were obtained. Included were data from the District of Columbia as well as all 50 states. A similar approach has also been applied in other research articles that have made use of the CDC WONDER database [11]. HHD was shown to be the primary cause of death in all cases or to be a contributing factor. Because deidentified data were made available to the public, institutional review board approval was not required for this study. The observational study reported in accordance with STROBE criteria.

### Data abstraction

The population count, the year and location of death, demographics, geographic distribution, state-specific data, and the distinction between urban and rural areas are all included in the retrieved data. Hospitals, houses, hospices, nursing homes, and long-term care institutions were among the places where people died. The demographic variables included age, race, ethnicity, and gender. Race and ethnicity were divided into the following categories: non-Hispanic White individuals, non-Hispanic Black or African-American individuals, non-Hispanic Latino individuals, non-Hispanic American Indian or Alaska Native individuals, non-Hispanic Asian individuals, and non-Hispanic Pacific Islanders. The National Centre for Health Statistics Urban–Rural Classification Scheme was used in the 2013 US Census to classify the population into urban areas [12]. These areas comprise large metropolitan areas with a population of one million or more, as well as medium/small metropolitan areas with a population ranging from fifty thousand to nine hundred ninety-nine thousand. The remainder of the population—less than 50,000—was categorized as rural. Based on predetermined criteria, the US Census Bureau divided the Northeast, Midwest, South, and West into four geographic divisions [13].

### Statistical analysis

The mortality rates per 100,000 individuals were calculated between 1999 and 2020 in order to examine regional variations in the death rate related to heart disease. The year, gender, race/ethnicity, state, and metropolitan/non-metropolitan status were used to categorize the rates. Additionally, the matching 95% confidence intervals (CIs) were given. The total number of deaths associated with HHD for each relevant year was divided by the corresponding US population to arrive at the crude mortality rates. Crude mortality rates were included alongside age-adjusted mortality rates (AAMRs) to provide a comprehensive view of HHD burden across different populations, enabling direct comparisons without age standardization. The number of HHD-related deaths in the US population in 2000 was adjusted to determine age-adjusted mortality rates or AAMR [14]. We computed the age-adjusted mortality rate (AAMR) annual percent change (APC) and its accompanying 95% confidence interval (CI) using the Joinpoint Regression Programme (Version 5.0.2, National Cancer Institute). The purpose of this analysis was to look at national trends in mortality from HHD on an annual basis [15, 16]. This method looks for meaningful changes in AAMR over time using log-linear regression models. The Age-Period-Cohort (APC) values were categorized as increasing or decreasing if, according to a two-tailed t test, the slope of the mortality change significantly deviated from zero. A two-tailed test was employed to assess the possibility of both increases and decreases in HHD mortality rates, ensuring a balanced analysis of the data trends. Given the large sample size, statistical significance was adjusted to *p* ≤ 0.01 to account for potential type I errors in the analysis.

## Results

Among individuals 55 and older, HHD was a contributing factor in 1,186,183 deaths between 1999 and 2020 (Supplemental Table 1). For 1,183,358 cases, the site of death was available for data (99.8%). 363,144 (30.7%) of these fatalities happened in hospitals, 199,032 (16.8%) in long-term care or nursing homes, 505,366 (42.7%) at home, 33,552 (2.8%) in hospice care, and the remaining 82,264 (7.0%) deaths happened in other places (Supplemental Table 2).

### HHD-related yearly patterns in AAMR

**Fig. 1:**
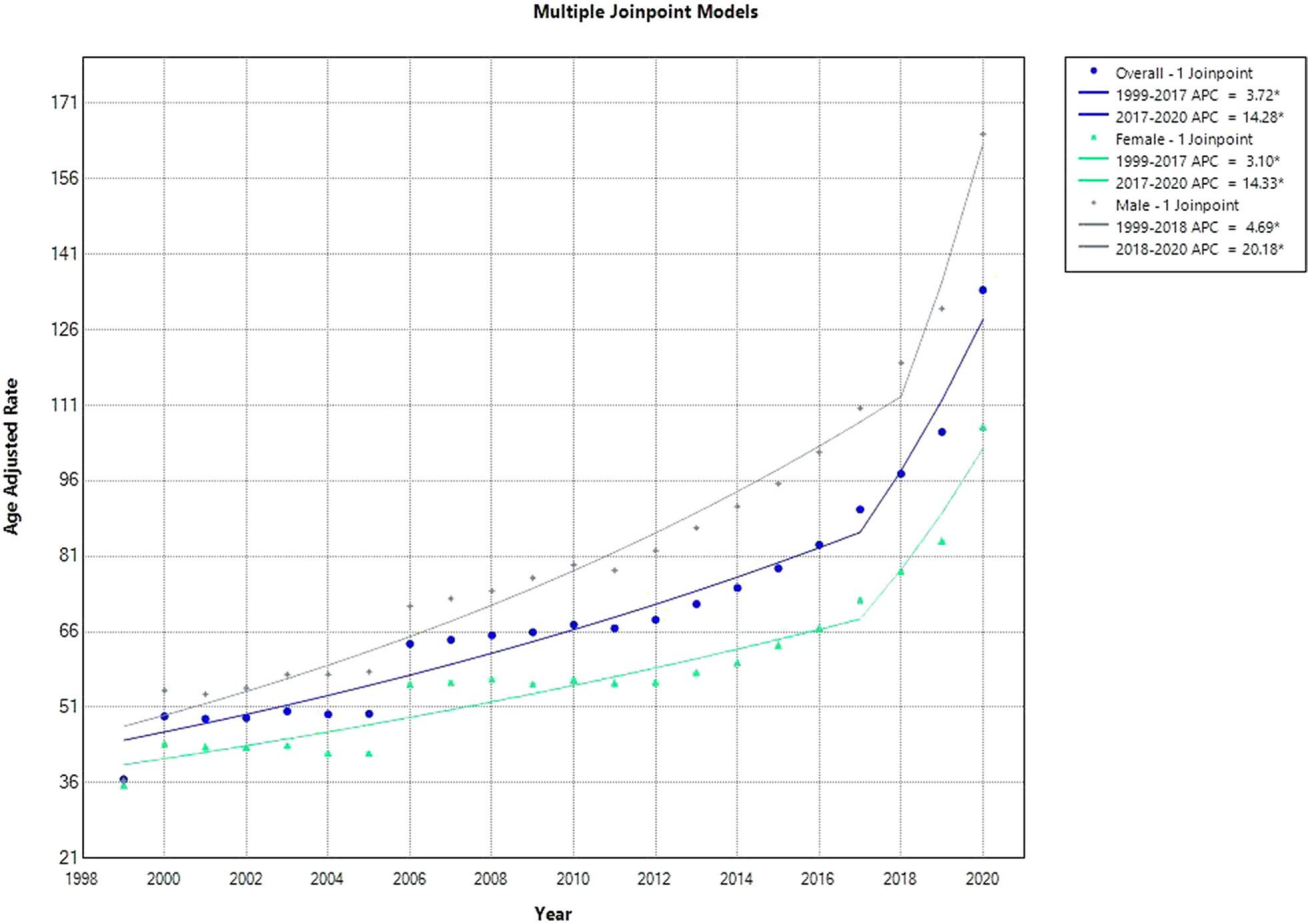
Overall and Sex-Stratified HHD-Related AAMRs per 100,000 in the USA, 1999–2020

By 2020, the AAMR for deaths in older individuals from heart disease (HDD) had increased from 36.7 in 1999 to 133.9. The AAMR increased steadily between 1999 and 2017, with an APC of 3.72 (95% [CI]: 2.72 to 4.49). An enormous spike between 2017 and 2020—with an APC of 14.28 (95% CI 8.21 to 22.93)—followed this (Supplemental Tables 3 and 4, Fig. 1).

### HHD-related yearly patterns in AAMR graded by gender

The overall AAMRs for males were 87.11 (95% CI 86.9–87.4), and women’s overall AAMRs were 59.8 (95% CI 59.6–59.9) across the duration of the study, with older males continuously having higher AAMRs than older women.

With an increase to 119.4 (95% CI 118.3–120.5) in 2018, the AAMR for older males was 36.4 (95% CI 35.6–37.2) in 1999 and showed an APC of 4.69 (95% CI 3.68 to 5.47). Subsequently, the pattern demonstrated an additional noteworthy increase from 2018 to 2020, with an APC of 20.18 (95% CI 9.54 to 25.30).

AAMRs for older girls, which showed a rise of 3.10 APC (95% CI 1.90 to 3.80) in 2017, were similar to older guys. In 1999, their AAMR was 35.6 (95% CI 35.0–36.2), and in 2017, it was 72.41 (95% CI 71.7–73.2). A further notable increase occurred between 2017 and 2020, resulting in an AAMR of 106.8 (95% CI 105.9–107.6), which corresponded to 14.33 APC (95% CI 7.12 to 24.39) (Fig. 1, Supplemental Tables 3 and 4).

### HHD-related yearly patterns in AAMR graded by race/ethnicity

**Fig. 2:**
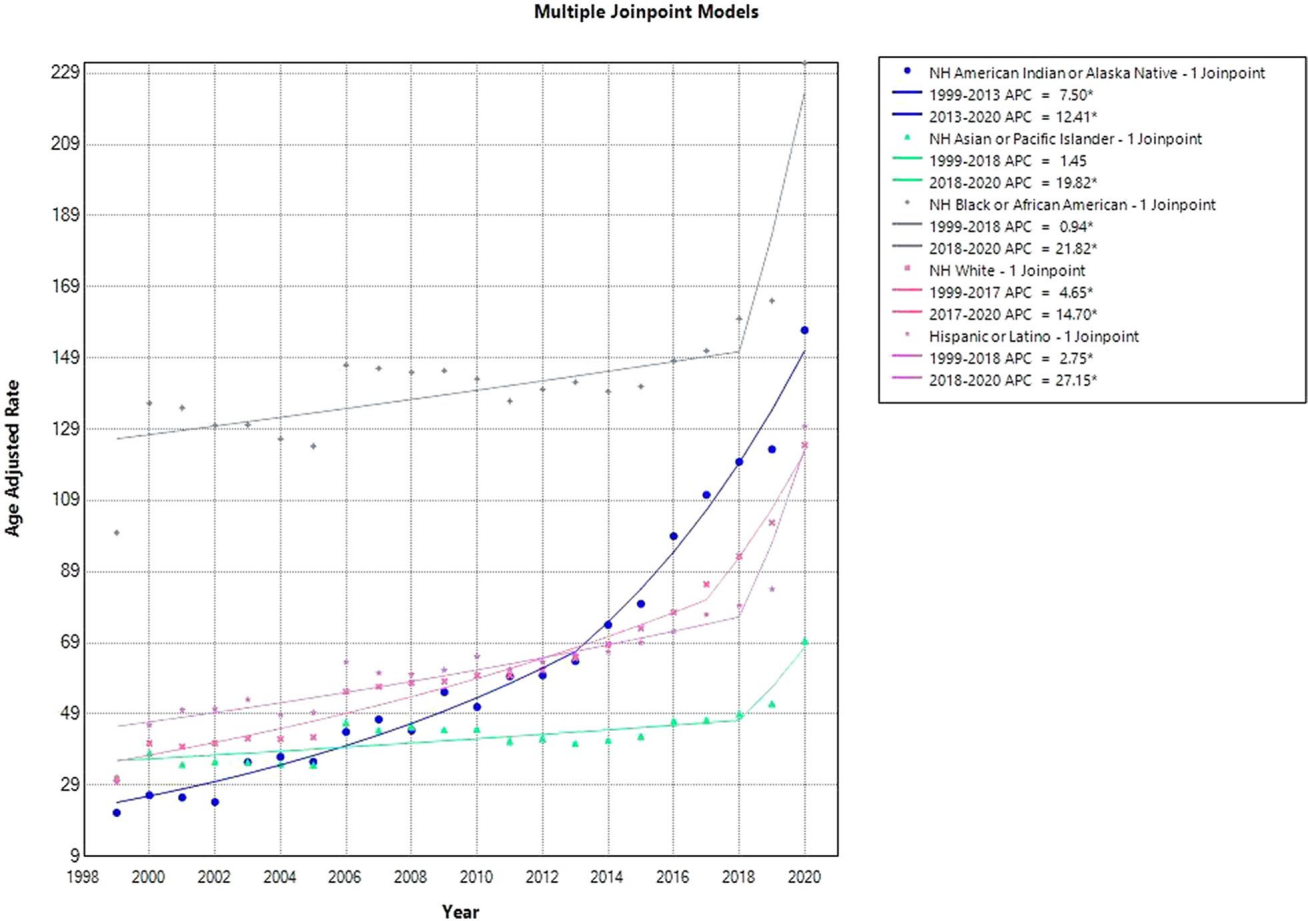
HHD-Related AAMRs per 100,000 Stratified by Race in the USA, 1999 to 2020. * Indicates that the APC is significantly different from zero at *α* = 0.05. NH: Non-Hispanic

With a mean AAMR of 147.4 (95% CI 146.8–148.1), non-Hispanic (NH) Black or African-American populations were the highest, followed by NH Asian or Pacific Islander groups at 45.6 (95% CI 45.0–46.1), NH White at 65.1 (95% CI 65.0–65.3), and NH American Indian or Alaska Native at 73.61 (95% CI 71.7–75.5). (Supplemental Tables 3 and 5, Fig. 2).

From 1999 to 2018, there was a little increase in the trends for NH Black or African-American, with an APC of 0.94 (95% CI 0.11 to 1.64). Then, from 2018 to 2020, there was a notable rise in AAMR, with an APC of 21.82 (95% CI 9.66 to 28.21). Similar patterns were seen in other racial groups as well, with an early rise in AAMRs followed by a notable jump.

Between 1999 and 2013, there was a significant increase in NH American Indian or Alaska Native populations’ AAMR, with an APC of 7.50 (95% CI 0.84 to 9.36) and a substantial surge of APC 12.41 (95% CI 10.29 to 19.54). The APC of 2.75 (95% CI 1.76 to 3.70) for Hispanic or Latino AAMRs increased from 1999 to 2018, and an even greater increase of APC 27.15 (95% CI 14.27 to 34.00) was seen from 2018 to 2020.

APC of 4.65 (95% CI 3.46 to 5.43) for NH White populations revealed an increase in AAMR between 1999 and 2017 and 14.70 (95% CI 8.15 to 23.51) for NH White populations between 2017 and 2020. APC of 1.45 (95% CI − 0.29 to 2.48), followed by another notable increase of APC 19.82 (95% CI 5.41 to 26.95), was the AAMR for the NH Asian or Pacific Islander group from 1999 to 2018 (Fig. 2, Supplemental Tables 3 and 5).

### Annual trends in HHD-related AAMR stratified by geographic region

#### The USA

**Fig. 3:**
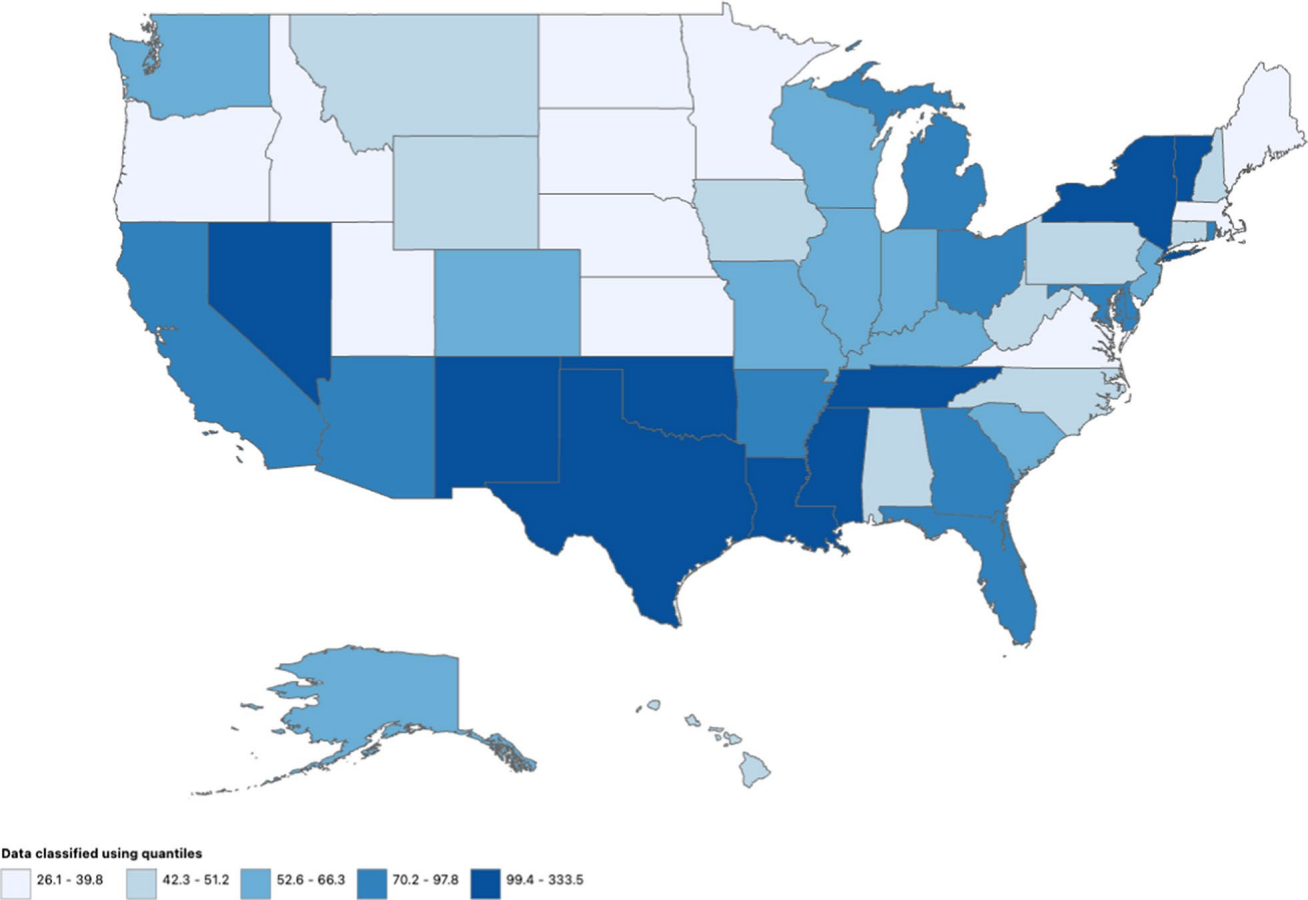
HHD-related AAMRs per 100,000 Stratified by State in the USA, 1999 to 2020

The states’ AAMR scores varied from 26.1 (95% CI 25.1–27.1) to 333.5 (95% CI 326.8–340.2). States with the highest AAMR that fell within the top 90th percentile were Mississippi, Oklahoma, Nevada, Vermont, and the District of Columbia. Nebraska, Oregon, North Dakota, Maine, and Minnesota were among those in the lowest 10th percentile (Fig. 3, Supplemental Table 6).

#### US census region

The South region (AAMR: 82.5; 95% CI 82.2–82.7) had the highest average mortality during the study period. The West (AAMR: 69.0; 95% Cl: 68.7–69.2), the Northeast (AAMR: 68.8; 95% CI 68.5–69.1), and the Midwest (AAMR: 64.0; 95% CI 63.7–64.2) had the next highest average mortality.

**Fig. 4:**
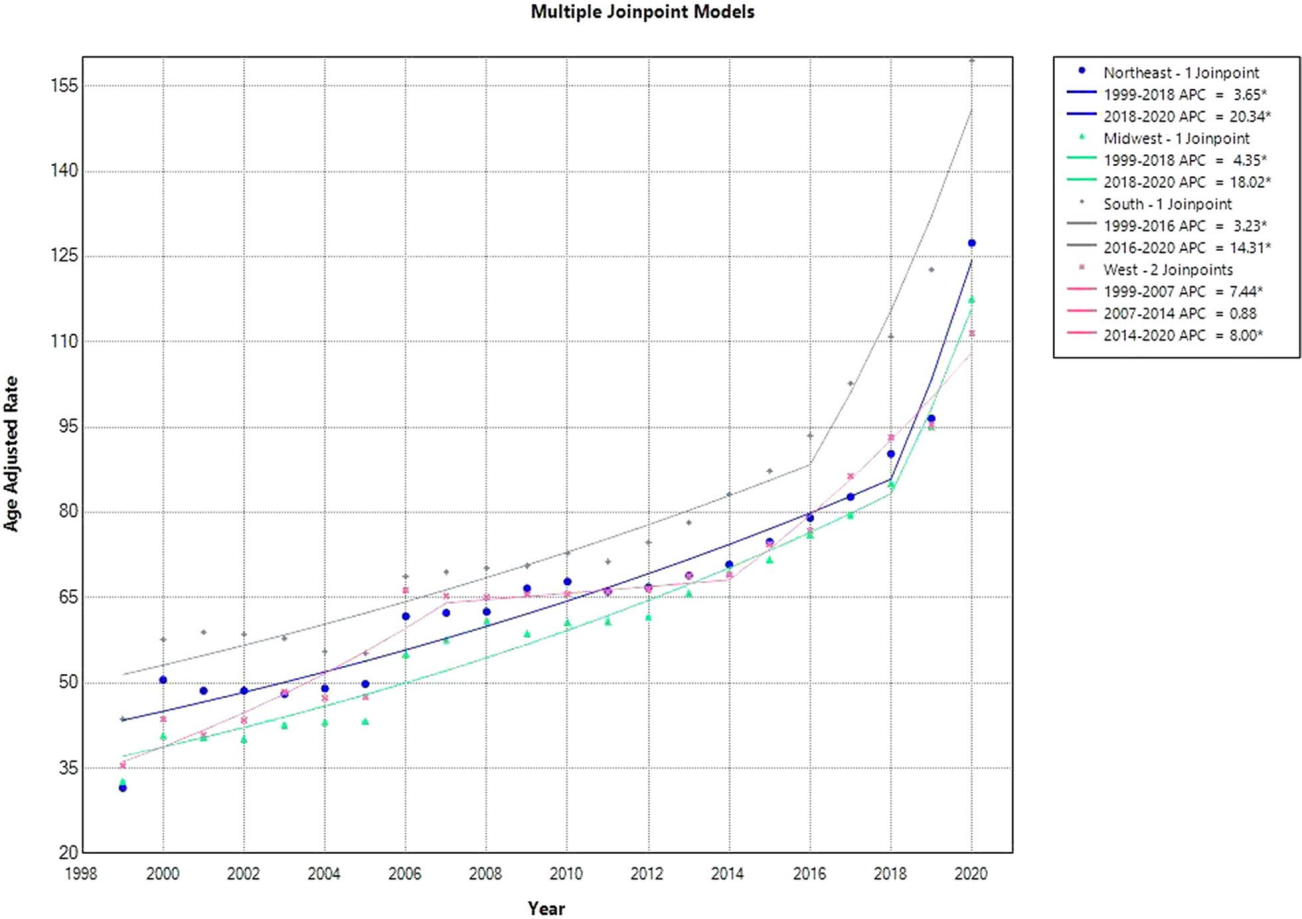
HHD-Related AAMRs per 100,000 Stratified by Census Region in the USA, 1999 to 2020. * Indicates that the APC is significantly different from zero at *α* = 0.05

The AAMR for the South area increased between 1999 and 2016, with an APC of 3.23 (95% CI 1.95 to 4.21), and between 2016 and 2020, there was a notable increase in APC 14.31 (95% CI 9.13 to 26.85). Between 1999 and 2007, the West region’s AAMR increased initially, with an APC of 7.44 (95% CI 5.62 to 11.80). After that, there was an additional spike in APC 0.88 (95% CI − 4.33 to 2.75) from 2007 to 2014. Ultimately, an APC of 8.00 (95% CI 6.27 to 11.66) indicates a considerable increase in AAMR from 2014 to 2020. From 1999 to 2018, the Northeast region’s AAMR increased, with an APC of 3.65 (95% CI 2.30 to 4.44). From 2018 to 2020, there was a significant spike in AAMR, with an APC of 20.34 (95% CI 6.90 to 27.06). Last but not least, the Midwest region’s AAMR increased initially between 1999 and 2018 with an APC of 4.35 (95% CI 3.39 to 4.94), and then, it spiked again between 2018 and 2020 with an APC of 18.02 (95% CI 7.47 to 23.38) (Fig. 4, Supplemental Tables 3 and 7).

#### Urbanization

**Fig. 5:**
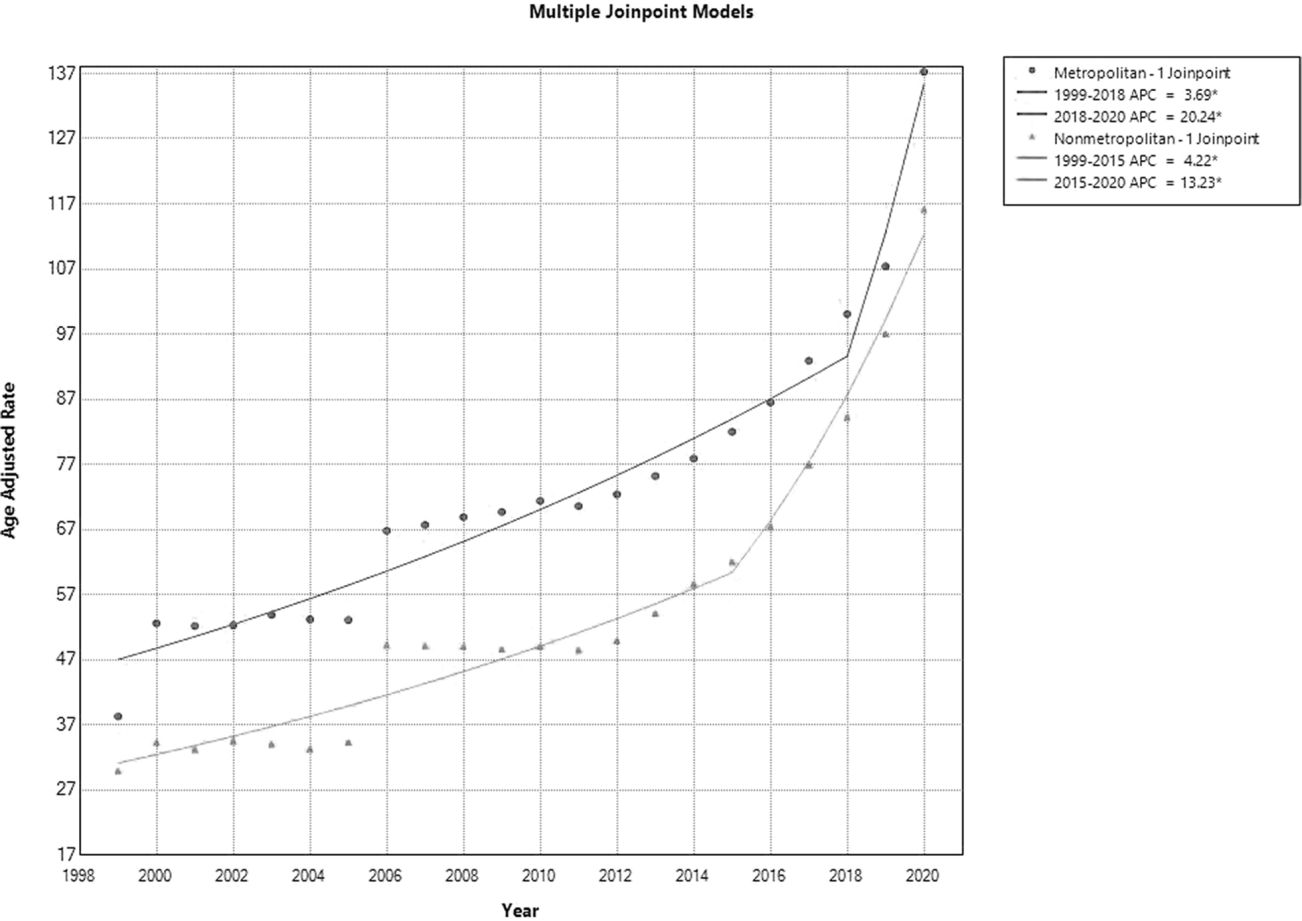
HHD-Related AAMRs per 100,000 Stratified by Urbanization in the USA, 1999 to 2020. * Indicates that the APC is significantly different from zero at *α* = 0.05

Compared to non-metropolitan areas (AAMR: 56.4; 95% CI 56.1–56.7), AAMR in metropolitan areas was generally higher (76.1; 95% CI 76.0–76.3). From 1999 to 2018, AAMR in urban areas climbed initially, with an APC of 3.69 (95% CI 2.84 to 4.37). Next, a rise with an APC of 20.24 (95% CI 9.04 to 25.88) was noted between 2018 and 2020. The AAMR for non-metropolitan areas showed a similar pattern, with a notable increase in APC of 13.23 (95% CI 9.54 to 22.66) from 2015 to 2020, following an initial rise in AAMR of APC 4.22 (95% CI 2.79 to 5.30) from 1999 to 2015 (Fig. 5, Supplemental Tables 3 and 8).

## Discussion

In this study assessing the trends in hypertensive heart disease (HHD) in older persons between 1999 and 2020, we report several key findings. The age-adjusted mortality rate (AAMR) was first widely elevated about HHD. From 1999 to 2017, the rates showed a consistent growing trend; however, from 2017 to 2020, there was a noticeable increase. In addition, it was noted that older men’s age-adjusted mortality rates (AAMRs) were consistently higher than older women’s. Thirdly, the populations with the highest age-adjusted mortality rates (AAMRs) were those who identified as non-Hispanic Black or African-American; these groups were followed by NH White, NH Asian or Pacific Islander, NH American Indian or Alaska Native, and Hispanic or Latino. Based on regional disparities, the age-adjusted mortality rates (AAMRs) were highest in the South, West, Northeast, and Midwest, respectively. In general, AAMRs were higher in metropolitan areas than in non-metropolitan ones. Understanding these trends is crucial to drive targeted interventions and healthcare policy, which in turn will improve outcomes for the senior population and lessen the burden of HHD.

According to our analysis, there were two separate times when AAMR significantly increased: from 1999 to 2017 and again from 2017 to 2020. In order to address the rising mortality burden linked to HHD, this biphasic pattern highlights the necessity for ongoing surveillance and intervention efforts. It also shows changing epidemiological dynamics. Numerous causes, including the aging population [17], the rising prevalence of hypertension [18], and inadequate management of cardiovascular risk factors [19, 20], could be to blame for the overall increase in AAMR throughout the study period. Higher reported death rates throughout this time may have resulted from improvements in HHD detection and diagnosis brought about by advancements in medical technology and healthcare delivery [21]. The most prominent rise in AAMR was observed during the COVID-19 pandemic when healthcare systems were severely affected, limiting access to care for patients with chronic conditions like hypertension and HHD. Delayed or reduced access to medical services during the pandemic exacerbated cardiovascular risks in elderly patients, who often have multiple comorbidities. A study by Wadhera et al. (2020) showed that there was an increase in cardiovascular mortality, particularly in patients with underlying conditions like hypertension, during the pandemic due to interruptions in routine healthcare services and delayed care-seeking behavior​ [22]. Further, the interaction between COVID-19 and cardiovascular diseases has been explored, with emerging evidence that the virus itself may have worsened cardiovascular conditions, contributing to increased mortality [23]. In case of future pandemics, and because elderly patients often face challenges in visiting doctors in person, there should be a focus on enhancing remote care solutions for elderly patients with HHD. Implementing telemonitoring systems such as wearable heart monitors or blood pressure monitors with Wi-Fi connectivity will allow healthcare providers to intervene early and adjust treatments as needed without requiring physical visits. Moreover, expanding telemedicine services will enable regular virtual consultations, ensuring that medication adjustments and management of symptoms can continue uninterrupted.

Throughout the study period, our data showed that older males had higher AAMRs than older women, even though women are less likely to seek medical attention for cardiovascular symptoms, which might delay diagnosis and treatment [24]. Men are more likely than women to have modifiable risk factors for hypertension, such as diabetes, smoking, and heavy alcohol use, which may raise their risk [18, 25–28]. To further explain the rising risk, men also have a larger genetic propensity to hypertension [29]. The gender gap in HHD mortality highlights the significance of gender-specific methods for clinical management and prevention of HHD.

One of the factors that could influence health outcomes in HHD is socioeconomic status (SES). Individuals with lower SES are more likely to face significant barriers to healthcare access, adherence to antihypertensive medication, and lifestyle management, which contribute to poor hypertension control and the progression to HHD. Havranek et al. (2015) emphasized that socioeconomic barriers, including financial hardship, limited access to primary care, and lower health literacy, are critical determinants of cardiovascular health, especially in conditions like hypertension which can then progress to HHD [30]. For elderly populations, these barriers often lead to delayed diagnosis or suboptimal management of hypertension, increasing the risk of severe cardiovascular outcomes and contributing to the high mortality rates associated with HHD. To tackle the impact of SES on HHD mortality, solutions must focus on enhancing access to care and improving the management of hypertension in low-income elderly populations. One potential approach is the establishment of low-cost hypertension management programs that provide subsidized medications and regular health checkups. These programs can be supported through partnerships between healthcare providers, local governments, and non-profit organizations to make hypertension treatments more affordable for elderly patients on fixed incomes. Lastly, mobile health units that bring healthcare services directly to underserved communities could play a critical role in providing regular blood pressure screenings and HHD management support to elderly individuals who lack access to transportation or local healthcare facilities.

Racial and ethnic disparities play a crucial role in HHD mortality, particularly the disproportionately higher rates observed in non-Hispanic Black individuals. These disparities can be understood through structural racism, which creates systemic barriers to accessing high-quality healthcare. Non-Hispanic Black populations are more likely to experience delayed diagnoses, poor management of hypertension, and a higher burden of comorbidities such as diabetes, all of which exacerbate HHD risk​ [31]. In addition, non-Hispanic Black individuals often require different antihypertensive treatment approaches due to genetic differences in response to standard therapies, which may not be widely recognized or addressed in many healthcare settings​ [32]. Therefore, the higher mortality rates in non-Hispanic Black elderly populations reflect not only individual health behaviors but also deeply entrenched systemic inequities that limit access to effective care. These differences show how important it is to develop culturally sensitive interventions and policies that target the underlying causes of HHD mortality in underserved populations. Furthermore, disparity in death rates may also be attributed to the rising prevalence of non-traditional cardiovascular risk factors, such as subclinical inflammatory markers like homocysteine, C-reactive protein, elevated low-density lipoprotein oxidation, lipoprotein (a), adiponectin, and plasminogen activator inhibitor-1 [33]. These characteristics are especially important when it comes to associated illnesses like heart failure and diabetes mellitus [9, 34]. Furthermore, in minority communities, cultural pressures and low trust in the healthcare system may result in delayed medical attention seeking and poor treatment regimen adherence [20].

The intricate relationship between social determinants of health and local healthcare systems is further underscored by the geographic inequities observed in HHD-related mortality. States with the highest AAMRs—the District of Columbia, Oklahoma, Nevada, Vermont, and Mississippi—face unique challenges stemming from a combination of inconsistent outpatient cardiology practices, variable adherence to guideline-based therapies, and the differing impacts of state Medicaid policies [9]. For instance, in states with limited Medicaid expansion, like Mississippi, access to preventive cardiovascular care is restricted, contributing to higher HHD mortality​. Additionally, poor management of hypertension and a high burden of comorbidities, such as diabetes and obesity, further exacerbate the risk of severe cardiovascular outcomes in these regions. Thus, to reduce HHD-related mortality, these regions might profit from focused state-wide public health programs. The significance of taking urbanization into account as a driver of HHD-related mortality is highlighted by the higher AAMRs seen in metropolitan areas as opposed to non-metropolitan locations. On the other hand, the total rates of cardiovascular death are significantly greater in rural than in metropolitan areas. Reducing the burden of HHD in both urban and non-metropolitan settings requires targeted interventions that improve cardiovascular health promotion, access to healthcare services, and address social determinants of health.

Several limitations must be taken into account. Firstly, underreporting of comorbid diseases and possible misclassification of deaths connected to HHD could induce biases in the data derived from death certificates. Secondly, the comprehensive understanding of the burden of hypertension among older adults is limited due to the exclusion of nonfatal events and long-term functional consequences. Thirdly, the reporting of deaths attributable to heart failure may be impacted by differences in healthcare use and access to preventive care, especially in underprivileged groups. The accuracy and dependability of our conclusions could be further impacted by variations in coding standards and data quality throughout jurisdictions. Finally, our study’s retrospective approach, which makes use of secondary data sources, naturally restricts our capacity to prove causation or draw conclusions about the direct causal linkages between treatments and observed mortality rates.

## Conclusion

The HHD-related AAMR increased steadily between 1999 and 2017, and then, it significantly increased between 2017 and 2020. Older males were found to have higher AAMRs than older females. The populations that were Black or African-American in New Hampshire as well as those in the states of Mississippi, Oklahoma, Nevada, Vermont, and the District of Columbia had the highest AAMRs. Moreover, it was discovered that the AAMRs in the Southern USA and its metropolitan areas were greater than those in other regions. The study underscores the necessity for tailored public health interventions, particularly in high-risk populations, to address the growing burden of HHD mortality. These findings emphasize the need for continued surveillance and adaptive strategies to mitigate the impact of HHD, especially in underserved communities.

## Supplementary Information

Supplementary file 1.

## Data Availability

The datasets analyzed during the current study are available in the CDC WONDER (https://wonder.cdc.gov/) repository.

## Abbreviations

CDC WONDER: Centers for Disease Control and Prevention
HHD: Hypertensive heart disease
AAMRs: Age-adjusted mortality rates
NH: Non-Hispanic
IHD: Ischemic heart disease
CI: Confidence intervals
APC: Annual percent change

## References

[1] Centers for Disease Control and Prevention (CDC) (1999) Decline in deaths from heart disease and stroke–United States, 1900–1999. MMWR Morb Mortal Wkly Rep 48(30):649–656 10488780

[2] Writing Group Members, Mozaffarian D, Benjamin EJ et al (2016) Heart disease and stroke statistics-2016 update: a report from the American heart association. Circulation. 10.1161/CIR.0000000000000350 10.1161/CIR.0000000000000350 26673558

[3] Santos M, Shah AM (2014) Alterations in cardiac structure and function in hypertension. Curr Hypertens Rep 16(5):428. 10.1007/s11906-014-0428-x 24639061 10.1007/s11906-014-0428-x PMC4051201

[4] Drazner MH (2011) The progression of hypertensive heart disease. Circulation 123(3):327–334. 10.1161/CIRCULATIONAHA.108.845792 21263005 10.1161/CIRCULATIONAHA.108.845792

[5] Tackling G, Borhade MB (2024) Hypertensive heart disease. StatPearls Publishing. Accessed March 25, 2024. https://www.ncbi.nlm.nih.gov/books/NBK539800/30969622

[6] Chan II (2024) Interpreting the observed sex differences in hypertensive heart disease burden. Eur J Prev Cardiol 31(1):21–22. 10.1093/eurjpc/zwad276 37607268 10.1093/eurjpc/zwad276

[7] Centers for disease control and prevention (CDC). Hypertension cascade: hypertension prevalence, treatment and control estimates among US adults aged 18 years and older applying the criteria from the American college of cardiology and American heart association’s 2017 hypertension guideline—NHANES 2017–2020. Accessed March 25, 2024. https://millionhearts.hhs.gov/data-reports/hypertension-prevalence.html.

[8] Kirkland EB, Heincelman M, Bishu KG et al (2018) Trends in healthcare expenditures among US adults with hypertension: national estimates, 2003–2014. J Am Heart Assoc. 10.1161/JAHA.118.008731 29848493 10.1161/JAHA.118.008731 PMC6015342

[9] Siddiqi TJ, Khan Minhas AM, Greene SJ et al (2022) Trends in heart failure-related mortality among older adults in the United States from 1999–2019. JACC Heart Fail 10(11):851–859. 10.1016/j.jchf.2022.06.012 36328654 10.1016/j.jchf.2022.06.012

[10] Castro-Porras LV, Rojas-Martínez R, Aguilar-Salinas CA, Bello-Chavolla OY, Becerril-Gutierrez C, Escamilla-Nuñez C (2021) Trends and age-period-cohort effects on hypertension mortality rates from 1998 to 2018 in Mexico. Sci Rep 11(1):17553. 10.1038/s41598-021-96175-0 34475436 10.1038/s41598-021-96175-0 PMC8413460

[11] Centers for disease control and prevention. CDC WONDER [Database]. Accessed March 25, 2024. https://wonder.cdc.gov/controller/datarequest/D77;jsessionid=CBB7708644CDF18DCBA4BAB1EEB1

[12] Aggarwal R, Chiu N, Loccoh EC, Kazi DS, Yeh RW, Wadhera RK (2021) Rural-urban disparities: diabetes, hypertension, heart disease, and stroke mortality among black and white adults, 1999–2018. J Am Coll Cardiol 77(11):1480–1481. 10.1016/j.jacc.2021.01.032 33736831 10.1016/j.jacc.2021.01.032 PMC8210746

[13] Ingram DD, Franco SJ (2014) 2013 NCHS urban-rural classification scheme for counties. Vital Health Stat 2(166):1–73 24776070

[14] Rethy L, Shah NS, Paparello JJ, Lloyd-Jones DM, Khan SS (2020) Trends in hypertension-related cardiovascular mortality in the United States, 2000 to 2018. Hypertension 76(3):e23–e25. 10.1161/HYPERTENSIONAHA.120.15153 32654559 10.1161/HYPERTENSIONAHA.120.15153 PMC9390965

[15] Anderson RN, Rosenberg HM (1998) Age standardization of death rates: implementation of the year 2000 standard. Natl Vital Stat Rep 47(3):1–16 9796247

[16] Joinpoint Regression Program. Published online March 2021. Accessed March 25, 2024. https://surveillance.cancer.gov/joinpoint/

[17] Fermini B, Bell DC (2022) On the perspective of an aging population and its potential impact on drug attrition and pre-clinical cardiovascular safety assessment. J Pharmacol Toxicol Methods 117:107184. 10.1016/j.vascn.2022.107184 35618160 10.1016/j.vascn.2022.107184

[18] Ostchega Y, Fryar CD, Nwankwo T, Nguyen DT (2020) Hypertension prevalence among adults aged 18 and over: United States, 2017–2018. NCHS Data Brief 364:1–8 32487290

[19] Basu S, Berkowitz SA, Phillips RL, Bitton A, Landon BE, Phillips RS (2019) Association of primary care physician supply with population mortality in the United States, 2005–2015. JAMA Intern Med 179(4):506–514. 10.1001/jamainternmed.2018.7624 30776056 10.1001/jamainternmed.2018.7624 PMC6450307

[20] Smedley BD, Stith AY, Nelson AR (2003) Unequal treatment: confronting racial and ethnic disparities in health care*.* National Academies Press (US) 25032386

[21] Wamble DE, Ciarametaro M, Dubois R (2019) The effect of medical technology innovations on patient outcomes, 1990–2015: results of a physician survey. J Manag Care Spec Pharm 25(1):66–71. 10.18553/jmcp.2018.18083 29927346 10.18553/jmcp.2018.18083 PMC10398270

[22] Wadhera RK, Shen C, Gondi S, Chen S, Kazi DS, Yeh RW (2021) Cardiovascular deaths during the COVID-19 pandemic in the United States. J Am Coll Cardiol 77(2):159–169. 10.1016/j.jacc.2020.10.055 33446309 10.1016/j.jacc.2020.10.055 PMC7800141

[23] Bandyopadhyay D, Akhtar T, Hajra A et al (2020) COVID-19 pandemic: cardiovascular complications and future implications. Am J Cardiovasc Drugs 20(4):311–324. 10.1007/s40256-020-00420-2 32578167 10.1007/s40256-020-00420-2 PMC7310596

[24] Möller-Leimkühler AM (2007) Gender differences in cardiovascular disease and comorbid depression. Dialogues Clin Neurosci 9(1):71–83. 10.31887/DCNS.2007.9.1/ammoeller 17506227 10.31887/DCNS.2007.9.1/ammoeller PMC3181845

[25] National center for health statistics. Percentage of current cigarette smoking for adults aged 18 and over, United States, 2019—2020. National Health Interview Survey. Accessed March 24, 2024. https://wwwn.cdc.gov/NHISDataQueryTool/SHS_adult/index.html

[26] National center for health statistics. Interactive Summary Health Statistics for Adults 2022. Accessed March 24, 2024. https://wwwn.cdc.gov/NHISDataQueryTool/SHS_adult/index.html

[27] National center for health statistics. Table 26. Normal weight, overweight, and obesity among adults aged 20 and over, by selected characteristics: United States, selected years 1988–1994 through 2015–2018. Accessed March 24, 2024. https://www.cdc.gov/nchs/data/hus/2019/026-508.pdf

[28] Centers for disease control and prevention. behavioral risk factor surveillance system. Accessed March 24, 2024. https://www.cdc.gov/brfss/

[29] Connelly PJ, Currie G, Delles C (2022) Sex Differences in the prevalence, outcomes and management of hypertension. Curr Hypertens Rep 24(6):185–192. 10.1007/s11906-022-01183-8 35254589 10.1007/s11906-022-01183-8 PMC9239955

[30] Havranek EP, Mujahid MS, Barr DA et al (2015) Social determinants of risk and outcomes for cardiovascular disease. Circulation 132(9):873–898. 10.1161/CIR.0000000000000228 26240271 10.1161/CIR.0000000000000228

[31] Calvin R, Winters K, Wyatt SB, Williams DR, Henderson FC, Walker ER (2003) Racism and cardiovascular disease in African Americans. Am J Med Sci 325(6):315–331. 10.1097/00000441-200306000-00003 12811228 10.1097/00000441-200306000-00003

[32] Flack JM, Sica DA, Bakris G et al (2010) Management of high blood pressure in blacks. Hypertension 56(5):780–800. 10.1161/HYPERTENSIONAHA.110.152892 20921433 10.1161/HYPERTENSIONAHA.110.152892

[33] Osei K, Gaillard T (2017) Disparities in cardiovascular disease and type 2 diabetes risk factors in blacks and whites: dissecting racial paradox of metabolic syndrome. Front Endocrinol (Lausanne) 8:204. 10.3389/fendo.2017.00204 28912752 10.3389/fendo.2017.00204 PMC5583515

[34] Rosenstock S, Whitman S, West JF, Balkin M (2014) Racial disparities in diabetes mortality in the 50 most populous US cities. J Urban Health 91(5):873–885. 10.1007/s11524-013-9861-4 24532483 10.1007/s11524-013-9861-4 PMC4199450

