# Supplementary material for: Trends in hypertensive heart disease-related mortality among older adults in the USA: a retrospective analysis from CDC WONDER between 1999 and 2020
Source: Egypt Heart J. 2025 Mar 4;77:27. doi: 10.1186/s43044-025-00622-6 (PMC11880464; doi:10.1186/s43044-025-00622-6)
Supplement: Supplementary file 1 [file 43044_2025_622_MOESM1_ESM.docx]

**Supplemental Table 1** Hypertensive Heart Disease–related deaths stratified by sex and race in Older Adults in the United States, 1999 to 2020

| **Hypertensive Heart Disease-Related Deaths** | | | | | | | | | |
| --- | --- | --- | --- | --- | --- | --- | --- | --- | --- |
| **Year** | **Overall** | **Women** | **Men** | **NH White** | **NH Black or African American** | **NH Asian or Pacific Islander** | **NH American Indian or Alaska Native** | **Hispanic or Latino** | **Population** |
| 1999 | 21356 | 13029 | 8327 | 15271 | 4762 | 368 | 46 | 784 | 58575867 |
| 2000 | 29001 | 16049 | 12952 | 20309 | 6613 | 486 | 65 | 1321 | 59266437 |
| 2001 | 29178 | 16033 | 13145 | 20146 | 6687 | 507 | 64 | 1548 | 60395586 |
| 2002 | 29958 | 16173 | 13785 | 20915 | 6588 | 528 | 66 | 1633 | 62225539 |
| 2003 | 31363 | 16532 | 14831 | 21930 | 6749 | 575 | 97 | 1809 | 63872474 |
| 2004 | 31547 | 16184 | 15363 | 22155 | 6749 | 588 | 111 | 1775 | 65508623 |
| 2005 | 32446 | 16469 | 15977 | 22869 | 6834 | 626 | 112 | 1873 | 67291295 |
| 2006 | 42405 | 22619 | 19786 | 30507 | 8259 | 885 | 135 | 2463 | 69094220 |
| 2007 | 44069 | 23221 | 20848 | 31886 | 8469 | 906 | 148 | 2515 | 70954145 |
| 2008 | 45861 | 23956 | 21905 | 33232 | 8660 | 979 | 148 | 2647 | 72934684 |
| 2009 | 47480 | 23995 | 23485 | 34049 | 9074 | 1008 | 193 | 2846 | 75028775 |
| 2010 | 49570 | 24760 | 24810 | 35669 | 9216 | 1064 | 189 | 3156 | 76750713 |
| 2011 | 50820 | 25250 | 25570 | 36678 | 9319 | 1076 | 231 | 3246 | 79456281 |
| 2012 | 53669 | 25842 | 27827 | 38416 | 9943 | 1178 | 249 | 3554 | 81731558 |
| 2013 | 57593 | 27322 | 30271 | 41297 | 10465 | 1243 | 279 | 3900 | 84020505 |
| 2014 | 61638 | 28790 | 32848 | 44443 | 10759 | 1370 | 356 | 4237 | 86320792 |
| 2015 | 66542 | 31162 | 35380 | 48109 | 11270 | 1515 | 394 | 4660 | 88638671 |
| 2016 | 72040 | 33349 | 38691 | 51959 | 12239 | 1745 | 497 | 5100 | 90707339 |
| 2017 | 79815 | 36956 | 42859 | 58108 | 12922 | 1878 | 585 | 5753 | 92854337 |
| 2018 | 87877 | 40496 | 47381 | 64332 | 14117 | 2022 | 666 | 6191 | 94703829 |
| 2019 | 97150 | 44382 | 52768 | 71765 | 14975 | 2273 | 727 | 6884 | 96506800 |
| 2020 | 124805 | 57002 | 67803 | 87703 | 21489 | 3181 | 944 | 10880 | 98063042 |
| **Total** | 1186183 | 579571 | 606612 | 851748 | 216158 | 26001 | 6302 | 78775 | 1694901512 |

NH: Non-Hispanic

**Supplemental Table 2** Hypertensive Heart Disease–related deaths stratified by place of death in older Adults in the United States, 1999 to 2020

| **Hypertensive Heart Disease-Related Deaths** | | | | | |  |
| --- | --- | --- | --- | --- | --- | --- |
| **Year** | **Medical Facility** | **Nursing Home/Long-term Care Facility** | **Hospice** | **Home** | **Other** |  |
|  |  |  |  |  |  |  |
| 1999 | 8659 | 5068 | 11 | 6815 | 809 |  |
| 2000 | 12192 | 5355 | 28 | 9975 | 1464 |  |
| 2001 | 11914 | 5405 | 200 | 10260 | 1591 |  |
| 2002 | 12203 | 5219 | 372 | 10835 | 1692 |  |
| 2003 | 12389 | 5385 | 572 | 11631 | 1889 |  |
| 2004 | 12087 | 5342 | 747 | 12042 | 1987 |  |
| 2005 | 12026 | 5417 | 740 | 12691 | 2042 |  |
| 2006 | 14716 | 8431 | 1068 | 16262 | 2516 |  |
| 2007 | 15421 | 8620 | 1222 | 16718 | 2660 |  |
| 2008 | 15604 | 8773 | 1536 | 17311 | 2592 |  |
| 2009 | 15557 | 8339 | 1782 | 18749 | 2839 |  |
| 2010 | 16433 | 8664 | 2169 | 20130 | 3250 |  |
| 2011 | 16381 | 8796 | 2769 | 20922 | 3475 |  |
| 2012 | 16723 | 8932 | 3008 | 22680 | 3777 |  |
| 2013 | 17197 | 9480 | 3432 | 24875 | 4231 |  |
| 2014 | 18056 | 9923 | 4062 | 26830 | 4602 |  |
| 2015 | 18638 | 10594 | 4639 | 29253 | 5233 |  |
| 2016 | 20067 | 10968 | 5195 | 32418 | 5558 |  |
| 2017 | 21185 | 12594 | 33552 | 36753 | 5816 |  |
| 2018 | 22006 | 13881 | 4095 | 41080 | 6831 |  |
| 2019 | 23634 | 15626 | 4559 | 45445 | 7789 |  |
| 2020 | 30056 | 18220 | 4571 | 61691 | 9621 |  |
| **Total** | 363144 | 199032 | 38230 | 505366 | 82264 |  |

**Supplemental Table 3** Annual percent change (APC) of Hypertensive Heart Disease-related age-adjusted mortality rates in older adults in the United States, 1999 to 2020

| **Year Interval** | **APC (95% CI)** |
| --- | --- |
| **Overall** | |
| 1999-2013 | -0.67 ( -2.49 to 1.80) |
| 2013-2018 | 2.62 (-1.35 to 4.52) |
| 2018-2020 | 9.81* (4.26 to 13.25) |
| **Male** | |
| 1999-2012 | -0.99*( -2.07 to -0.54) |
| 2012-2018 | 1.94 (-0.25 to 3.75) |
| 2018-2020 | 10.39* (5.74 to 13.44) |
| **Female** | |
| 1999-2015 | -0.89*(-1.43 to -0.43) |
| 2018-2020 | 6.03*(3.91 to 9.85) |
| **NH White** | |
| 1999-2012 | -0.67* (-1.94 to -0.16) |
| 2012-2018 | 2.32 (-0.18 to 4.09) |
| 2018-2020 | 10.50* (5.05 to 13.73) |
| **NH Black or African American** | |
| 1999-2014 | -0.36 (-1.30 to 0.39) |
| 2014-2020 | 6.78* (4.68 to 11.28) |
| **NH American Indian or Alaska Native** | |
| 1999 - 2003 | 4.34 (-1.28 to 26.18) |
| 2003-2013 | -2.11 (-11.17 to 5.25) |
| 2013-2020 | 3.62 (-1.02 to 12.79) |
| **Hispanic or Latino** | |
| 1999-2018 | 0.64 (-0.02 to 1.18) |
| 2018-2020 | 14.42* (5.61 to 18.94) |
| **NH Asian or Pacific Islander** | |
| 1999-2014 | -0.82 (-1.94 to 0.07) |
| 2014-2020 | 5.60 (3.62 to 10.64) |
| **Nonmetropolitan areas** | |
| 1999-2015 | -0.44* (-0.89 to -0.04) |
| 2015-2020 | 6.81* (4.97 to 9.63) |
| **Metropolitan area** | |
| 1999-2012 | -0.82* (-2.36 to -0.27) |
| 2012-2018 | 2.10 (-0.40 to 4.10) |
| 2018-2020 | 10.52* (5.30 to 13.78) |
| **Northeast region** | |
| 1999-2012 | -0.83 (-3.57 to 3.04) |
| 2012-2018 | 1.61 (-1.44 to 4.09) |
| 2018-2020 | 12.00* (4.64 to 16.49) |
| **Midwest region** | |
| 1999-2012 | -0.84* (-2.73 to -0.29) |
| 2012-2018 | 1.67 (-0.41 to 4.01) |
| 2018-2020 | 12.95* (7.26 to 16.90) |
| **South region** | |
| 1999-2014 | -0.37 (-0.91 to 0.10) |
| 2014-2020 | 5.72* (4.29 to 7.88) |
| **West region** | |
|  | |
| 1999-2014 | -0.99* (-1.42 to -0.60) |
| 2014-2020 | 3.82* (2.62 to 5.78) |

* Indicates statistical significance. AAMR: age-adjusted mortality rate; APC: annual percent change; NH: non-Hispanic;

**Supplemental Table 4** Overall and sex‐stratified Hypertensive Heart Disease–related age-adjusted mortality rates per 10,000 in older adults in the United States

| **Age-Adjusted Mortality Rate (95% CI)** | | | |
| --- | --- | --- | --- |
| **Year** | **Male** | **Female** | **Overall** |
| 1999 | 36.4 (35.6-37.2) | 35.6 (35-36.2) | 36.7 (36.2-37.2) |
| 2000 | 54.3 (53.3-55.2) | 43.8 (43.1-44.5) | 49.2 (48.6-49.8) |
| 2001 | 53.6 (52.7-54.5) | 43.2 (42.5-43.9) | 48.7 (48.1-49.2) |
| 2002 | 54.8 (53.9-55.7) | 43.1 (42.4-43.8) | 48.9 (48.4-49.5) |
| 2003 | 57.5 (56.6-58.4) | 43.5 (42.8-44.1) | 50.2 (49.7-50.8) |
| 2004 | 57.5 (56.6-58.5) | 41.9 (41.3-42.6) | 49.6 (49-50.1) |
| 2005 | 58.1 (57.1-59) | 41.9 (41.2-42.5) | 49.7 (49.1-50.2) |
| 2006 | 71.1 (70.1-72.2) | 55.6 (54.9-56.3) | 63.6 (63-64.2) |
| 2007 | 72.6 (71.6-73.6) | 56 (55.3-56.7) | 64.4 (63.8-65) |
| 2008 | 74.1 (73.1-75.1) | 56.6 (55.8-57.3) | 65.3 (64.7-65.9) |
| 2009 | 76.7 (75.7-77.7) | 55.7 (55-56.4) | 65.9 (65.3-66.5) |
| 2010 | 79.3 (78.3-80.3) | 56.5 (55.8-57.2) | 67.4 (66.8-68) |
| 2011 | 78.2 (77.2-79.2) | 55.9 (55.2-56.6) | 66.7 (66.1-67.3) |
| 2012 | 82.1 (81.1-83.1) | 56 (55.3-56.7) | 68.4 (67.8-69) |
| 2013 | 86.6 (85.6-87.6) | 58 (57.3-58.7) | 71.5 (70.9-72.1) |
| 2014 | 90.9 (89.9-92) | 59.9 (59.2-60.6) | 74.7 (74.1-75.3) |
| 2015 | 95.4 (94.4-96.4) | 63.4 (62.7-64.1) | 78.6 (78-79.2) |
| 2016 | 101.7 (100.6-102.7) | 66.8 (66.1-67.5) | 83.3 (82.6-83.9) |
| 2017 | 110.4 (109.4-111.5) | 72.4 (71.7-73.2) | 90.3 (89.6-90.9) |
| 2018 | 119.4 (118.3-120.5) | 78 (77.2-78.8) | 97.4 (96.8-98.1) |
| 2019 | 130.2 (129-131.3) | 84.1 (83.3-84.9) | 105.7 (105.1-106.4) |
| 2020 | 164.9 (163.6-166.2) | 106.8 (105.9-107.6) | 133.9 (133.1-134.6) |
| Total | 87.1 (86.9-87.4) | 59.8 (59.6-59.9) | 72.6 (72.5-72.8) |

**Supplemental Table 5** Hypertensive Heart Disease–related age-adjusted mortality rates per 100,000 stratified by race in the United States, 1999 to 2020.

| **Age-Adjusted Mortality Rate (95% CI)** | | | | | |
| --- | --- | --- | --- | --- | --- |
| **Year** | **NH White** | **NH Black or African American** | **NH American Indian or Alaska Native** | **Hispanic or Latino** | **NH Asian or Pacific Islander** |
| 1999 | 30.8 (30.3-31.3) | 100 (97.1-102.8) | 21.3 (15.4-28.8) | 29.9 (27.7-32.1) | 31.4 (28.1-34.8) |
| 2000 | 40.8 (40.2-41.3) | 136.3 (133-139.6) | 26.2 (20-33.7) | 45.9 (43.3-48.5) | 38.1 (34.6-41.6) |
| 2001 | 39.9 (39.4-40.5) | 135 (131.7-138.2) | 25.6 (19.4-33.1) | 50.2 (47.6-52.8) | 35 (31.8-38.1) |
| 2002 | 40.8 (40.3-41.4) | 130 (126.8-133.2) | 24.3 (18.5-31.2) | 50.5 (47.9-53) | 35.7 (32.6-38.9) |
| 2003 | 42.1 (41.6-42.7) | 130.2 (127-133.3) | 35.5 (28.4-43.8) | 53.1 (50.6-55.7) | 35.5 (32.5-38.5) |
| 2004 | 42 (41.4-42.5) | 126.2 (123.1-129.3) | 37 (29.6-44.4) | 48.8 (46.4-51.1) | 35.1 (32.2-38) |
| 2005 | 42.5 (41.9-43) | 124.2 (121.2-127.2) | 35.6 (28.5-42.7) | 49.4 (47.1-51.7) | 34.7 (31.9-37.5) |
| 2006 | 55.3 (54.7-55.9) | 147 (143.7-150.2) | 44 (36.1-51.9) | 63.6 (61-66.2) | 46.7 (43.6-49.9) |
| 2007 | 56.7 (56.1-57.3) | 146.1 (142.9-149.3) | 47.5 (39.3-55.7) | 60.6 (58.1-63) | 44.6 (41.6-47.6) |
| 2008 | 57.8 (57.2-58.5) | 145 (141.9-148.2) | 44.4 (36.8-52.1) | 60.2 (57.8-62.6) | 45.5 (42.6-48.4) |
| 2009 | 58.2 (57.5-58.8) | 145.4 (142.4-148.5) | 55.2 (46.8-63.6) | 61.4 (59.1-63.7) | 44.7 (41.9-47.6) |
| 2010 | 59.9 (59.3-60.6) | 143.1 (140.1-146.1) | 51 (43.2-58.9) | 65.2 (62.9-67.6) | 44.9 (42.1-47.6) |
| 2011 | 59.9 (59.3-60.6) | 136.9 (134.1-139.8) | 59.6 (51.4-67.9) | 61.6 (59.4-63.8) | 41.4 (38.9-44) |
| 2012 | 61.4 (60.8-62) | 140.1 (137.2-142.9) | 59.9 (52-67.9) | 63.6 (61.4-65.7) | 42.2 (39.8-44.7) |
| 2013 | 64.8 (64.2-65.5) | 142.2 (139.4-145) | 63.9 (55.9-71.9) | 65.7 (63.6-67.8) | 40.8 (38.5-43.1) |
| 2014 | 68.6 (68-69.3) | 139.6 (136.9-142.4) | 74.1 (65.9-82.3) | 66.5 (64.5-68.6) | 41.8 (39.5-44) |
| 2015 | 73.1 (72.4-73.7) | 141 (138.3-143.7) | 80 (71.6-88.4) | 69.1 (67-71.1) | 42.9 (40.7-45.1) |
| 2016 | 77.6 (77-78.3) | 148.2 (145.5-150.9) | 99 (89.8-108.2) | 72.3 (70.3-74.4) | 47.2 (45-49.5) |
| 2017 | 85.5 (84.8-86.2) | 151 (148.3-153.7) | 110.6 (101.2-120) | 77 (75-79.1) | 47.4 (45.2-49.6) |
| 2018 | 93.4 (92.6-94.1) | 160 (157.2-162.7) | 119.8 (110.3-129.4) | 79.6 (77.5-81.6) | 49.2 (47-51.4) |
| 2019 | 102.8 (102.1-103.6) | 165.1 (162.4-167.8) | 123.3 (114-132.7) | 84.2 (82.2-86.3) | 52 (49.9-54.2) |
| 2020 | 124.5 (123.7-125.3) | 231.8 (228.6-235) | 156.8 (146.4-167.1) | 129.8 (127.3-132.3) | 69.7 (67.3-72.2) |
| **Total** | 65.1 (65-65.3) | 147.4 (146.8-148.1) | 73.6 (71.7-75.5) | 69.8 (69.3-70.3) | 45.6 (45-46.1) |

NH: Non-Hispanic

**Supplemental Table 6** Hypertensive Heart Disease–related age-adjusted mortality rates per 100,000 stratified by state in older adults in the United States, 1999 to 2020.

| **State** | **Age-Adjusted Mortality Rate (95% CI)** |
| --- | --- |
| Alabama | 51.2 (50.3-52.1) |
| Alaska | 52.9 (49.8-56) |
| Arizona | 85.8 (84.8-86.8) |
| Arkansas | 86.8 (85.4-88.3) |
| California | 70.9 (70.5-71.3) |
| Colorado | 56.4 (55.4-57.4) |
| Connecticut | 42.4 (41.5-43.3) |
| Delaware | 75.1 (72.7-77.5) |
| District of Columbia | 333.5 (326.8-340.2) |
| Florida | 80.9 (80.4-81.4) |
| Georgia | 76.1 (75.3-77) |
| Hawaii | 43.3 (41.9-44.8) |
| Idaho | 38.5 (37.1-39.9) |
| Illinois | 66.3 (65.6-66.9) |
| Indiana | 53 (52.2-53.8) |
| Iowa | 45.6 (44.7-46.6) |
| Kansas | 36 (35.1-37) |
| Kentucky | 52.9 (52-53.9) |
| Louisiana | 124.5 (123-125.9) |
| Maine | 29.6 (28.5-30.8) |
| Maryland | 78.2 (77.2-79.2) |
| Massachusetts | 39.8 (39.2-40.5) |
| Michigan | 97.8 (96.9-98.6) |
| Minnesota | 31.3 (30.7-32) |
| Mississippi | 138.7 (136.8-140.6) |
| Missouri | 64.9 (64-65.7) |
| Montana | 42.3 (40.6-44) |
| Nebraska | 26.1 (25.1-27.1) |
| Nevada | 151.5 (149.3-153.8) |
| New Hampshire | 49 (47.4-50.6) |
| New Jersey | 61.3 (60.6-62) |
| New Mexico | 104.6 (102.7-106.6) |
| New York | 109 (108.4-109.6) |
| North Carolina | 47.8 (47.2-48.4) |
| North Dakota | 29.3 (27.7-31) |
| Ohio | 78.6 (77.9-79.3) |
| Oklahoma | 163.3 (161.5-165.1) |
| Oregon | 28.9 (28.2-29.6) |
| Pennsylvania | 42.4 (41.9-42.8) |
| Rhode Island | 70.2 (68.2-72.3) |
| South Carolina | 52.6 (51.7-53.6) |
| South Dakota | 33.4 (31.8-35) |
| Tennessee | 101.5 (100.4-102.6) |
| Texas | 99.4 (98.8-100) |
| Utah | 37 (35.8-38.2) |
| Vermont | 147.9 (144-151.9) |
| Virginia | 39.2 (38.6-39.8) |
| Washington | 64.3 (63.5-65.2) |
| West Virginia | 44.2 (43-45.4) |
| Wisconsin | 56.8 (56-57.6) |
| Wyoming | 43.1 (40.7-45.6) |

**Supplemental Table 7** Hypertensive Heart Disease –related age-adjusted mortality rates per 100,000 stratified by census region in older adults in the United States, 1999 to 2020

| **Census Region** | **Year** | **Age-Adjusted Mortality Rate (95% CI)** |
| --- | --- | --- |
| Northeast | 1999 | 13.2 (12.9-13.5) |
| Northeast | 2000 | 13.3 (13-13.6) |
| Northeast | 2001 | 13.2 (13-13.5) |
| Northeast | 2002 | 12.8 (12.5-13.1) |
| Northeast | 2003 | 13.2 (13-13.5) |
| Northeast | 2004 | 13.2 (12.9-13.5) |
| Northeast | 2005 | 13.4 (13.1-13.7) |
| Northeast | 2006 | 13.2 (12.9-13.5) |
| Northeast | 2007 | 13.3 (13-13.6) |
| Northeast | 2008 | 13.9 (13.6-14.1) |
| Northeast | 2009 | 13.3 (13-13.5) |
| Northeast | 2010 | 13.6 (13.3-13.9) |
| Northeast | 2011 | 14.0 (13.7-14.3) |
| Northeast | 2012 | 13.8 (13.5-14) |
| Northeast | 2013 | 13.9 (13.6-14.2) |
| Northeast | 2014 | 13.5 (13.2-13.7) |
| Northeast | 2015 | 14.1 (13.8-14.4) |
| Northeast | 2016 | 13.7 (13.4-14) |
| Northeast | 2017 | 13.9 (13.6-14.2) |
| Northeast | 2018 | 14.3 (14-14.6) |
| Northeast | 2019 | 14.6 (14.3-14.8) |
| Northeast | 2020 | 16.8 (16.5-17.1) |
| Northeast | **Total** | 13.7 (13.7-13.8) |
| Midwest | 1999 | 16.1 (15.8-16.4) |
| Midwest | 2000 | 15.8 (15.4-16.1) |
| Midwest | 2001 | 16.2 (15.9-16.5) |
| Midwest | 2002 | 16.1 (15.8-16.4) |
| Midwest | 2003 | 16.2 (15.9-16.5) |
| Midwest | 2004 | 15.5 (15.2-15.8) |
| Midwest | 2005 | 15.9 (15.6-16.2) |
| Midwest | 2006 | 15.8 (15.5-16.1) |
| Midwest | 2007 | 16.0 (15.7-16.3) |
| Midwest | 2008 | 16.5 (16.2-16.8) |
| Midwest | 2009 | 16.1 (15.8-16.4) |
| Midwest | 2010 | 16.7 (16.4-17) |
| Midwest | 2011 | 17.1 (16.8-17.4) |
| Midwest | 2012 | 16.4 (16.1-16.7) |
| Midwest | 2013 | 16.9 (16.6-17.2) |
| Midwest | 2014 | 17.0 (16.7-17.3) |
| Midwest | 2015 | 17.6 (17.3-17.9) |
| Midwest | 2016 | 17.6 (17.3-17.9) |
| Midwest | 2017 | 18.1 (17.8-18.4) |
| Midwest | 2018 | 18.2 (17.9-18.5) |
| Midwest | 2019 | 18.8 (18.5-19.1) |
| Midwest | 2020 | 21.9 (21.6-22.2) |
| Midwest | **Total** | 17.0 (16.9-17.1) |
| South | 1999 | 15.3 (15-15.5) |
| South | 2000 | 15.4 (15.2-15.7) |
| South | 2001 | 15.4 (15.1-15.6) |
| South | 2002 | 15.6 (15.3-15.8) |
| South | 2003 | 15.5 (15.3-15.7) |
| South | 2004 | 15.2 (14.9-15.4) |
| South | 2005 | 15.3 (15-15.5) |
| South | 2006 | 15.3 (15-15.5) |
| South | 2007 | 15.0 (14.8-15.2) |
| South | 2008 | 15.4 (15.2-15.6) |
| South | 2009 | 15.6 (15.3-15.8) |
| South | 2010 | 15.7 (15.5-15.9) |
| South | 2011 | 15.6 (15.4-15.8) |
| South | 2012 | 15.7 (15.5-15.9) |
| South | 2013 | 15.6 (15.3-15.8) |
| South | 2014 | 15.7 (15.5-15.9) |
| South | 2015 | 16.1 (15.9-16.3) |
| South | 2016 | 16.3 (16.1-16.5) |
| South | 2017 | 16.7 (16.5-16.9) |
| South | 2018 | 16.7 (16.5-16.9) |
| South | 2019 | 17.1 (16.9-17.3) |
| South | 2020 | 19.8 (19.6-20) |
| South | **Total** | 16.0 (15.9-16) |
| West | 1999 | 13.6 (13.3-13.9) |
| West | 2000 | 13.2 (12.9-13.5) |
| West | 2001 | 13.9 (13.6-14.2) |
| West | 2002 | 13.5 (13.2-13.8) |
| West | 2003 | 13.6 (13.3-13.9) |
| West | 2004 | 13.4 (13.1-13.7) |
| West | 2005 | 13.5 (13.2-13.8) |
| West | 2006 | 13.7 (13.4-14) |
| West | 2007 | 13.9 (13.6-14.2) |
| West | 2008 | 14.2 (13.9-14.5) |
| West | 2009 | 14.2 (13.9-14.4) |
| West | 2010 | 14.7 (14.4-15) |
| West | 2011 | 14.9 (14.6-15.2) |
| West | 2012 | 14.8 (14.5-15) |
| West | 2013 | 15.0 (14.7-15.3) |
| West | 2014 | 15.2 (14.9-15.5) |
| West | 2015 | 15.7 (15.4-16) |
| West | 2016 | 16.3 (16-16.5) |
| West | 2017 | 16.6 (16.4-16.9) |
| West | 2018 | 16.8 (16.5-17) |
| West | 2019 | 17.1 (16.9-17.4) |
| West | 2020 | 20.1 (19.8-20.4) |
| West | **Total** | 15.1 (15.1-15.2) |
| **Total** | **Total** | 15.6 (15.6-15.6) |

**Supplemental Table 8** Hypertensive Heart Disease–related age-adjusted mortality rates per 100,000 stratified by urban-rural classification in older adults in the United States, 1999 to 2020

| **Age-Adjusted Mortality Rate (95% CI)** | | |
| --- | --- | --- |
| **Year** | **Metropolitan** | **Nonmetropolitan** |
| 1999 | 14.4 (14.2-14.5) | 16.2 (15.8-16.5) |
| 2000 | 14.3 (14.1-14.4) | 16.2 (15.9-16.6) |
| 2001 | 14.4 (14.3-14.6) | 16.6 (16.3-17) |
| 2002 | 14.3 (14.2-14.5) | 16.3 (16-16.7) |
| 2003 | 14.4 (14.2-14.5) | 16.8 (16.4-17.2) |
| 2004 | 14.1 (13.9-14.2) | 16.3 (16-16.7) |
| 2005 | 14.2 (14.1-14.4) | 16.5 (16.1-16.8) |
| 2006 | 14.3 (14.1-14.4) | 16.2 (15.8-16.5) |
| 2007 | 14.3 (14.1-14.4) | 16.4 (16.1-16.8) |
| 2008 | 14.7 (14.5-14.8) | 17.2 (16.8-17.5) |
| 2009 | 14.5 (14.4-14.6) | 17.1 (16.7-17.4) |
| 2010 | 14.9 (14.8-15.1) | 17.1 (16.7-17.4) |
| 2011 | 15.0 (14.9-15.2) | 17.6 (17.3-18) |
| 2012 | 14.8 (14.7-15.0) | 17.6 (17.3-18) |
| 2013 | 15.0 (14.9-15.1) | 17.6 (17.2-17.9) |
| 2014 | 14.9 (14.8-15.0) | 18.2 (17.8-18.5) |
| 2015 | 15.4 (15.3-15.6) | 18.7 (18.3-19) |
| 2016 | 15.5 (15.4-15.7) | 19.1 (18.7-19.4) |
| 2017 | 15.9 (15.8-16.1) | 19.1 (18.8-19.5) |
| 2018 | 16.1 (15.9-16.2) | 19.4 (19.1-19.8) |
| 2019 | 16.4 (16.3-16.6) | 20.2 (19.8-20.5) |
| 2020 | 19.0 (18.9-19.2) | 23.5 (23.1-23.9) |
| Total | 15.2 (15.1-15.2) | 17.8 (17.7-17.9) |
